# Role of Oral Nutritional Supplements Enriched with B-hydroxy-B-Methylbutyrate in Maintaining Muscle Function and Improving Clinical Outcomes in Various Clinical Settings

**DOI:** 10.1007/s12603-018-0995-7

**Published:** 2018-01-04

**Authors:** Alejandro Sanz-Paris, M. Camprubi-Robles, J. M. Lopez-Pedrosa, S. L. Pereira, R. Rueda, M. D. Ballesteros-Pomar, J. M. Garcia Almeida, A. J. Cruz-Jentoft

**Affiliations:** 1 Nutrition Unit, Universitary Hospital Miguel Servet, Isabel the Catholic 1-3, Zaragoza, 50009 Spain; 2 Abbott Nutrition, Research and Development, Granada, Spain; 3 Abbott Nutrition, Research & Development, Columbus, OH USA; 4 Complejo Asistencial Universitario de León, León, Spain; 5 Hospital Virgen de la Victoria de Málaga, Málaga, Spain; 6 Geriatric Department, Hospital Universitario Ramón y Cajal, Instituto Ramón y Cajal de Investigación Sanitaria (IRYCIS), Madrid, Spain

**Keywords:** Sarcopenia, malnutrition, oral nutritional supplements, disease, aging, HMB, muscle wasting

## Abstract

Aging and disease-related malnutrition are well associated with loss of muscle mass and function. Muscle mass loss may lead to increased health complications and associated increase in health care costs, especially in hospitalized individuals. High protein oral nutritional supplements enriched with β-hydroxy-β-methylbutyrate (HP-ONS+HMB) have been suggested to provide benefits such as improving body composition, maintaining muscle mass and function and even decreasing mortality rates. The present review aimed to examine current evidence on the effect of HP-ONS+HMB on muscle-related clinical outcomes both in community and peri-hospitalization patients. Overall, current evidence suggests that therapeutic nutrition such as HP-ONS+HMB seems to be a promising tool to mitigate the decline in muscle mass and preserve muscle function, especially during hospital rehabilitation and recovery.

## Introduction

Maintaining skeletal muscle mass and function is important for sustaining health through the lifespan. Muscle comprises about 30–40 % of the body weight and is considered a key tissue for physical movement and posture, as well as for vital functions including chewing, swallowing, and breathing (1). Besides its relevant role in structural maintenance of the body, muscle has been recognized as an important active metabolic and homeostatic organ acting as the main reservoir for proteins and serving as the main tissue for glucose disposal by the body (2). Given these essential roles of muscle in overall well-being, it is not surprising that decline in muscle mass and function leads to increased risk of mobility-disability (3), infections or even mortality (4).

**Figure 1:**
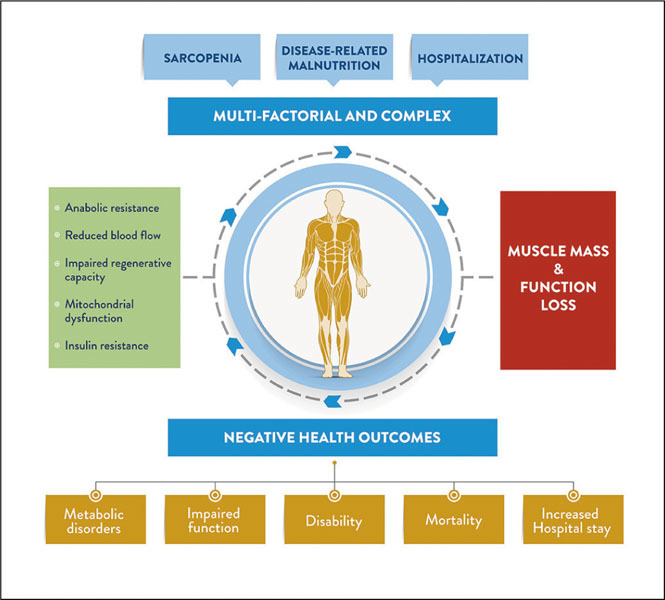
Common causes and consequences of muscle mass and function loss

During the last decade, cumulative research has been focused on the clinical causes and consequences of muscle mass and strength loss due to aging and/or associated with pathological conditions in older adults. Muscle loss combined with strength or functional loss is currently defined as ‘Sarcopenia’ (5). The European working group on sarcopenia in older people has suggested sub-categories of sarcopenia based on cause. This includes “primary” sarcopenia due to age-related muscle loss and “secondary” sarcopenia that comprises; a) disuse-related sarcopenia as a result of hospitalization or physical inactivity, b) disease-related sarcopenia that includes acute loss of muscle mass and strength due to acute or chronic disease, and c) nutrition-related sarcopenia resulting from inadequate dietary intake of energy and/or protein (5). Multiple contributing factors are involved in loss of muscle mass. For instance, progressive muscle decline can be a consequence of aging-related muscle dysfunction such as anabolic resistance, insulin resistance, reduced blood flow, impaired regenerative capacity, as well as mitochondrial dysfunction (6) (Fig. 1). Finally, some endocrine mechanisms such as thyroid dysfunction and reduced anabolic hormones may also lead to muscle loss. Muscle loss has been associated with negative health outcomes such as augmented risk of falls, which is associated with potential fractures, and impaired activities of daily living (ADLs) (3), metabolic disorders, negative hospitalization outcomes such as infections, health complications and increased length of stay (LOS), mobility-disability, and even mortality (7, 8) (Fig. 1). Even, it has been suggested that potential differences may exist in effects of low muscle relative to either body mass or height on cardiometabolic health in middle-aged and older adults (9).

Muscle loss can also occur as a result of malnutrition, when nutritional intake is not optimal resulting in both ‘over’ or ‘under’ nutrition. Of special interest is disease related malnutrition (DRM) that refers to ‘under nutrition’ characterized by a deficit of energy, protein and other nutrients, loss of appetite, and disease-related catabolism. Additionally, it is known that impaired immunity because of malnutrition predisposes to infections and decreases the ability of the body to recover from infections. Thus, the combined effect of DRM and loss of muscle mass due to hospitalization-related immobilization can cause severe functional decline (10), which has a negative impact on subsequent clinical and economic outcomes, such as high rate of non-elective hospital readmission (8, 11).

DRM is a frequent problem in all health care settings, including hospitals, care homes, and sheltered housing being high prevalent in older individuals and in rehabilitation settings (12). Therefore, lifestyle interventions that address both malnutrition and associated-muscle loss is of great importance.

Several approaches have been evaluated as potential strategies to mitigate muscle loss, such as exercise and/or therapeutic nutrition, and pharmaceutical interventions such as myostatin inhibitors, testosterone and selective androgen receptor modulators. Resistance exercise has well established benefits on preserving muscle (13). Thus, exercise in combination with therapeutic nutrition has also shown some promising results as an effective approach to target muscle loss in older adults (13).

Oral nutritional supplements (ONS) containing high levels of proteins, essential amino acids, and branched chain amino acids have also been reported to support muscle mass maintenance in different patient settings (14), although the optimal levels to meet patients’ needs remain uncertain (15). In particular, ingestion of protein sources enriched with Leucine (Leu) is thought to offer benefits in the preservation of muscle mass and function during aging and illness (14, 16).

Of special relevance is a Leu metabolite, β-hydroxy-β-methylbutyrate (HMB), which exerts its effects through protective, anticatabolic mechanisms, and has been shown to directly influence protein synthesis (17) and mitochondrial dynamics in skeletal muscle (18). HMB has been shown to benefit muscle mass and strength in various clinical populations such as chronic obstructive pulmonary disease (COPD), cancer cachexia, acquired immune deficiency syndrome, sepsis, and endotoxemia (17, 19, 20). Recently, HMB has been added to high protein ONS as a means to target muscle loss in context of an optimized nutrition supplement that provides macro and micronutrients to meet the daily requirements of patients with nutritional insufficiencies/deficiencies. Administration of these high protein HMB-enriched oral nutritional supplements (HP-ONS+HMB) has shown promise in helping adults maintain muscle mass and function in hospital and community settings (4, 21-23). Therefore, the purpose of this review is to summarize and discuss the current evidence that examined nutritional support, mainly through HP-ONS+HMB on muscle function and clinical outcomes in adults with, or at risk of, malnutrition, and both in community and peri-hospitalization settings.

## Methods

PubMed was used to search for relevant articles from the index date to August 2017. Keywords used in the search are summarized as follows: ‘HMB’ or ‘beta-hydroxy-beta-methylbutyrate’ and (oral nutrition supplements, enteral nutritional supplementation, nutritional intervention, replacement, therapy, treatment, effects or administration) and (muscle building, muscle breakdown, muscle function, muscle strength, grip, muscle loss, muscle wasting, or sarcopenia, bed rest, disuse atrophy, or malnutrition, disease related malnutrition) and (body composition, free fat mass, fat mass, muscle mass, lean body mass) and (amino acid supplementation, amino acid metabolite) and (aged, aging, older or elderly).

## Role of ONS in clinical practice

Sarcopenia and malnutrition frequently coexist especially in older adults who are hospitalized or those with chronic disease (DRM). The coexistence of both conditions can further aggravate disease prognosis and negatively impact clinical outcomes. Malnutrition is a condition that often goes unrecognized especially in the hospital although, it has been associated with negative clinical and economical outcomes such as increased LOS, health costs, complications, readmission rates, and mortality rates (8). In fact, although malnutrition prevalence may be very high ranging from 8% to 62%, depending on clinical setting and population type, in an eleven-year retrospective study, which analyzed data from 44 million adult impatient episodes, only 1.6% of the population received ONS intervention (24).

Prevention and treatment of malnutrition include dietary advice to increase the protein and energy content of the diet, food fortification, or nutrient supplementation via ONS (25, 26). In clinical practice, this is usually addressed by adding commercially available ONS to standard diet after systematic assessment of the nutritional status and nutritional requirements (energy, proteins and micronutrients) of the patients to ensure an optimized intervention to address specific nutritional needs (27, 28).

Evidence is still limited on the clinical effectiveness of dietary advice (29). Also results from nutritional interventions studies in malnourished populations are mixed possibly due to variations in study design, inclusion criteria, and composition of the nutritional intervention (27), factors that may have contributed to the failure of some studies to show significant effects. In addition, many nutrition intervention studies target hospital outcomes such as LOS, episode cost, and readmission probability which requires large sample size, which is not always reachable (24).

However, a number of systematic reviews and meta-analyses consistently indicate clinical benefits of using ONS on outcomes, including dietary intake, body composition, disease complications, mortality and LOS (30-33).

On the other hand, a cumulative body of evidence suggests that ONS enriched with specific nutrients, and administrated as a sole source of nutrition or in addition to normal diet could have a positive impact on adults under pathological conditions, especially among hospitalized patients. In fact, several recent systematic reviews and meta-analysis have shown a potential beneficial effect of ONS, especially those containing high protein levels (with ≥20% of total energy from protein; HP-ONS), on clinical outcomes in elderly people at risk of malnutrition (34, 35). Protein is key for driving muscle anabolism and provides the amino acid building blocks needed to rebuild muscle (36). Muscle protein synthesis is significantly diminished in the elderly and chronic disease populations (37). Based on current reviewed evidence on the benefits of protein intake in treating age-related decline in muscle mass, strength, and functional abilities, the European society for clinical nutrition and metabolism recently increased the protein recommendations for healthy elderly (1-1.2 g protein/Kg body weight/day) and malnourished or at risk of malnutrition elderly (1.2-1.5 g protein/Kg body weight/day) (38). Therefore, dietary interventions consisting of HP-ONS during sarcopenia and illness-associated muscle wasting have been currently proposed as potential nutritional strategies to mitigate muscle loss and related outcomes (16, 34). In this line, a meta-analysis including 36 randomized control trials (RCTs) (n=3,790) revealed that a range of clinical, functional and nutritional benefits and economic implications was favored in patients at hospital or community setting (mean age 74 years) receiving HP-ONS with no loss of appetite or reduction in normal food intake as compared to control group (35). A major finding was a significant overall reduction (19%) in a range of complications together with significant increases in hand grip strength (HGS), mean total energy, protein intakes, body weight, and mid-arm muscle circumference. Interestingly, HP-ONS was found to be effective in improving muscle strength with 5 out of 6 RCTs reporting significant mean changes in HGS that were greater in the HP-ONS group than the control after meta-analysis (1.76 Kgf (95% Confidence Interval 0.36-3.17), p=0.014, n=219). These findings are consistent with conclusions from other systematic reviews including different types of HP-ONS (34, 39). However, long-term population studies are still needed to conclusively demonstrate the efficacy of HP-ONS in improving muscle and functional outcomes.

In conclusion, evidence for HP-ONS on mitigating sarcopenia outcomes is still weak (13).

## HMB alone or combined with other amino acids

Several systematic reviews and meta-analyses have evaluated the potential role of HMB supplementation, either alone or in combination with other amino acids, in improving muscle quality and function both in older adults with sarcopenia and under pathological conditions (14, 17, 40).

Recently, a systematic review of the clinical evidence focused on oral supplementation with amino acids and amino acid metabolites (included HMB) in patients under critical illness or other similar muscle wasting illness (COPD, chronic heart failure, age-related muscle wasting (sarcopenia) or disuse atrophy (bed-rest) was performed by Wandrag et al. (14). Results from four studies including intensive care unit patients revealed that despite the fact that the studies were classified as moderate and low quality with limitations, HMB supplementation seemed to improve nitrogen balance in patients, and the addition of Arginine (Arg) and/or Glutamine (Gln) appeared to counterbalance any benefit of HMB alone.

Beneficial effects were also reported in a recently published systematic review and meta-analysis of RCTs conducted by Wu et al. (40), which aimed to investigate whether HMB supplementation had significant effects on body composition and muscle strength in healthy older adults and those with pathological conditions with a mean age of ≥ 65 years. Six studies were identified to be included in the meta-analysis showing that supplementation of HMB alone or in combination with other amino acids increased muscle mass gain in the intervention groups compared to the control groups (standard mean difference=0.352 kg; 95% CI: 0.11, 0.594; Z value=2.85; p=0.004). Yet, there were no differences in fat mass changes between groups (standard mean difference=-0.08 kg, p=0.511). This review also found that out of the 9 RCTs that evaluated muscle strength and functionality outcomes in response to HMB, two of the studies (40, 41) reported improvements in the aforementioned outcomes. The effect of a 12 week supplementation with a combination of HMB (2 g/d), Arg (5 g) and Lysine (Lys) (1.5 g) in elderly sedentary women (~76.7 years) was examined by Flakoll et al. (41), which found that at the end of the study, HGS was significantly greater in the HMB-supplemented group than in the placebo group (p=0.04). Further, there was a 17% improvement in the get-up-and-go performance time in the HMB-supplemented group versus the placebo group (p=0.002).

**Table 1:**
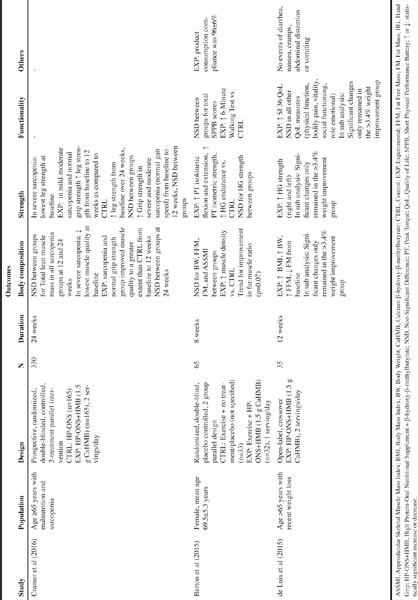
Summary of studies using HP-ONS+HMB in community dwelling older adults

A more recent study by Stout et al. (42) in older adults also found improvements in physical performance in response to HMB supplementation. This study was a double-blind, placebo controlled trial carried out in two phases. Each phase included 54 ambulatory males and females (aged >65 years). Phase I was a non-exercise phase where participants were supplemented with HMB (3 g/d) or placebo and phase II was the exercise phase where participants were supplemented with HMB (3 g/d) or placebo in combination with progressive resistance training (RT) (3 days a week). For Phase I, body composition analysis revealed a significant increase from baseline in total and leg lean mass within the HMB group (but not placebo) at 3 and 6 months, with a trend (p=0.09) favoring HMB over placebo for leg lean mass at 6 months. Also, the HMB group had significantly greater leg strength and muscle quality than the placebo group at the end of 6 months and greater fat mass loss at 24 weeks. For Phase II, RT had a significant benefit on lean mass, strength and function in both groups, reinforcing the benefits of progressive RT. Thus, these findings supported a benefit of HMB on muscle mass, strength and muscle quality in the absence of RT, and reinforced the benefits of RT on improving body composition and muscle functionality.

Another previously published systematic review of randomized trials examined the clinical literature on the effectiveness of HMB supplementation in healthy and pathological conditions (17). Notably, the authors concluded that most of selected studies exhibited a beneficial effect of HMB either alone or in combination with other amino acids (Arg and Gln or Arg and Lys) in mitigating loss of muscle mass and function in both healthy older adults and in patients with different pathological conditions.

Several studies have examined the effect of HMB in combination with other amino acids on postoperative rehabilitation. Reduced muscle strength has been observed during postoperative recovery in patients with osteoarthritis after total knee arthroplasty. Nishizaki et al. (43) investigated the effect of supplementation with a combination of HMB (3g), Arg (14 g), and Gln (14 g) versus placebo control on postoperative recovery of quadriceps strength in patients aged 65–80 years (n=23) after total knee arthroplasty. Postoperative rehabilitation in these patients consisted of strength-training and range-of-motion exercises, starting in bed (postoperative day 1), followed by walking training on subsequent days of rehabilitation. Muscle strength (knee extension) of the operated and non-operated was measured on postoperative days 14, 28, and 42. Whereas the control group showed a significant loss of muscle strength on day 14 (p=0.02), the HMB group attenuated muscle strength decline for the operated-side knee over pre- and post-operative periods. There was no significant difference between groups in muscle cross sectional area (measured by computerized tomography) before surgery and 42 days post-surgery. The duration of rehabilitation was similar for both groups, with a comparable LOS (19.1±3.7 days for the HMB vs. 18.9±3.3 days for the control), but there was a trend for weight loss over time for the HMB group (p=0.06). These findings are clinically relevant since patients who undergo total knee arthroplasty have been reported to have a 20% to 30% decline in muscle strength and walking ability, even at 1 year after surgery. Therefore, preservation of leg strength could lead to an improved walking ability thereby enhancing ADLs and improving quality of life (QoL).

Studies showing muscle inactivity due to extended bed-rest (e.g. during hospitalization) have shown to induce rapid muscle atrophy and loss of strength and power especially in the elderly. Healthy older subjects have been reported to lose ~1 kg of lean tissue from the lower extremities after 10 days of bed-rest, with an associated ~16% decline in isokinetic knee extensor strength [44]. Loss of muscle mass is a key factor in the elderly in relation to loss of functional capacity, as well as hospital morbidity and mortality. Deutz et al. (45) recently evaluated the effect of HMB (3 g/d) versus placebo control in healthy older adults (n=19) put on 10 days of complete bed-rest. Bed-rest caused a significant decrease in total LBM (2.05 ± 0.66 kg; p=0.02, paired t-test) in the control group, whereas treatment with HMB prevented this decline over bed-rest (0.17 ±0.19 kg; p=0.23, paired t-test). A statistically significant difference (p=0.02, ANOVA) between treatment groups for change in LBM over bed-rest was reported.

Altogether, these findings suggest that supplementation of HMB alone or in combination with other amino acids may be a valuable nutritional therapy to help maintain muscle mass and functionality in healthy older adults or hospitalized patients as well as during recovery/rehabilitation.

## HMB-enriched ONS in community dwelling older adults

The associated loss of muscle mass with aging has been recognized as a major health concern related to a decline in physical function, lower QoL and mortality. Recent publications have shown that supplementation with HP-ONS+HMB resulted in favorable muscle health outcomes in community dwelling older adults (Table 1).

Berton et al. (23) aimed at evaluating whether HP-ONS+HMB given as 1 serving/day for 8 weeks together with a mild fitness program could improve physical performance, muscle strength and body composition parameters. This open labeled RCT was conducted in a group of healthy community dwelling women (aged ≥ 65 years) attending a mild fitness program including mainly aerobic exercise and some resistance exercise. Participants in the control group were encouraged not to take any supplementation that increases their physical performance, however, diet specifications were not provided. No significant group differences were observed over 8 weeks for the primary outcomes such as short physical performance battery scores or balance test scores. The greatest benefits related to HP-ONS+HMB supplementation were observed for the secondary outcomes such as improved hand-grip endurance (delta=21.41±16.28 s; p=0.02) and peak torque (PT) isokinetic flexion (delta = 1.56±1.56 Nm; p=0.03), extension (delta=3.32±2.61 Nm; p=0.03) and PT isometric strength (delta=9.74±3.90 Nm; p=0.02), while no changes were observed for HGS. Notably, 6-minute walking test improved from the baseline to the follow-up (delta=7.67±8.29 m; p=0.04) in supplemented group. There were no significant changes in body weight (BW), fat free mass (FFM), abdominal fat mass (FM) or appendicular skeletal muscle mass index (ASMMI) between groups. However, HP-ONS+HMB group did show significant increases in muscle density at radius and tibia sites as compared to control (p=0.03), as well as a trend for improvement in fat: muscle ratio (p=0.07).Since SPPB at BL was >11 (fully functional), it is not surprising that this outcome did not change at the end of the intervention period, due to low sensitivity of the measure to detect minor changes in functional status. Overall, this study points to the beneficial effect of HP-ONS+ HMB in context of a mild fitness program on clinically relevant measures like isometric and isokinetic strength, and 6-minute walking test since impairment in these outcomes is mainly associated with fall risks, low bone mineral density, hip fractures, and functional limitations in ADLs.

Likewise, an open labeled study performed by de Luis et al. [46] investigated the effect of a HP-ONS+HMB (2 servings per day) on muscle strength and QoL in elderly patients (>65 years) with recent weight loss (>5% during previous 3 months) over a 12-week period. Overall, this study showed that the 35 patients receiving the HP-ONS+HMB reported significant benefits in anthropometric parameters such as increased body mass index (BMI), weight, and FFM (p>0.05) and increases in blood biochemical variables including prealbumin (1.5±4.1 mg/dl). The physical and general health domain, as part of the Short Form-36 Health Survey, significantly (p<0.05) improved, as well as the right and left HGS. Sub-analysis of two groups (‘greater than’ and ‘less than’ 3.4% weight improvement) revealed significant (p<0.05) improvements in body composition (2 Kg gain in lean mass), strength, and muscle function in the ‘greater than’ 3.4 % weight improvement’ subgroup. Notably, subjects with a greater weight gain (percentage of weight improvement >3.4%) consumed on average more HP-ONS+HMB (1.86+0.82 units/day) than subjects with a smaller weight gain (<3.4%) (1.25+0.78 units/day). This study however has some important limitations such as the open label design, the lack of an isocaloric control group, and a small sample size. Nevertheless, this study does demonstrate the clinical benefits of consuming HP-ONS+HMB in a population undergoing weight loss. It remains to be seeing if the observed benefits are due to caloric intake versus the specific composition of the ONS consumed.

A recent double-blinded RCT by Cramer et al. (47) evaluated the effects of two HP-ONS on malnourished and sarcopenic older adults. Subjects received 2 servings/day of HP-ONS+HMB or HP-ONS (control group) for 24 weeks. Overall, both ONS groups showed improvement in isokinetic PT, muscle quality, grip strength, and gait speed from baseline with no differences between groups. Similarly, both groups significantly increased leg strength from baseline over 24 weeks (p<0.001), with no treatment differences. However, when stratified for sarcopenia severity, early benefits (12 weeks) on leg strength was observed with HP-ONS+HMB supplementation over control in the non-severe sarcopenia group (normal grip strength) (p=0.032). In addition, in this group muscle quality improved to a greater extent than the control group (p=0.027) from baseline to 12 weeks. By 24 weeks, both groups showed improvements in leg strength and MQ with no treatment differences.

This study points to the overall benefit of supplementing malnourished-sarcopenic individuals with HP-ONS for improving muscle outcome, even in that absence of an exercise program. In addition, it shows that there are early benefits to be seen with supplementation of HP-ONS+HMB on mildmoderate sarcopenic subpopulations as early as 12 weeks into intervention. These results also point to the need for early intervention in order to achieve benefits on muscle-related outcomes in older sarcopenic adults.

Overall, these three studies indicate that in elderly community-dwelling patients the consumption of HP-ONS+HMB may be useful for improving muscle relatedfunctional outcomes and QoL. However, additional well controlled studies in a larger population are warranted to fully validate the findings of these published studies.

## HMB-enriched ONS in patients in peri-hospitalization setting

Use of HP-ONS+HMB can also lead to significant clinical, functional and nutritional benefits in individuals with, or at risk of, malnutrition in hospital setting. It is estimated that 30-50% of hospitalized patients are malnourished and even short LOS may have direct clinical consequences such as loss of LBM accompanied with an accelerated muscle function decline [10]. In addition, chronic diseases such as cancer and COPD can lead to severe muscle loss. Several published studies have been identified that examined the effect of HP-ONS+HMB on muscle function and metabolism in patients with different pathologies under peri-hospitalization conditions (Table 2).

It has been reported that adequate nutrition reduces immobilization and hospitalization in orthopedic patients with malnutrition through an improvement in wound healing, muscle mass and muscle strength (48). In a recent RCT, Ekinci et al. (21) evaluated the effects of HP-ONS+HMB over and above standard postoperative nutrition (experimental) versus standard postoperative nutrition alone (control) on muscle strength and mobilization in malnourished women aged 65–94 years who underwent hip fracture surgery. This study found that 2 servings per day of a HP-ONS+HMB, added to the standard postoperative nutrition plan for 30 days was able to significantly increase HGS as compared to control group, (8.63±3.83 Kgf vs 6.40±3.86 Kgf; p=0.026), but not muscle mass. In addition, would-healing period was significantly shorter in the experimental versus control group (14.56±2.80 days vs. 15.93±2.18 days; p<0.05) and the immobilization period was also reduced (81.3%; 26/32 of treated patients were mobile on day 30 vs. 26.7%; 8/30 in control group; p=0.001) without changes to BMI. However, hospitalization time was not significantly changed in the first 30 days and no significant differences were detected in C-reactive protein, arm circumference, triceps skinfold thickness, and calf circumference. Although important limitations included the lack of placebo control and assessment of dietary intake was based on the patient’s declaration, these results suggest that HP-ONS+HMB may benefit accelerated wound healing, increased mobilization and muscle strength in older adults after orthopedic surgery.

**Table 2:**
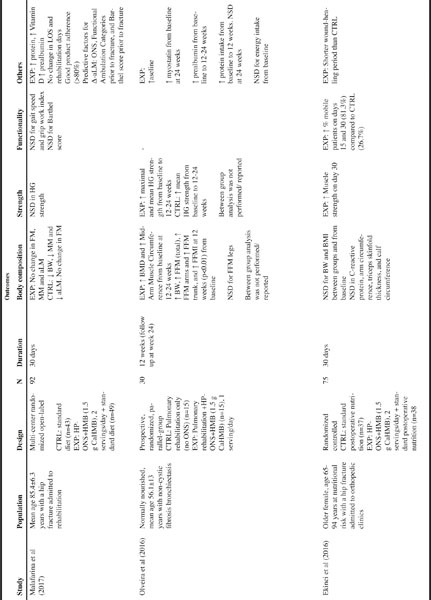

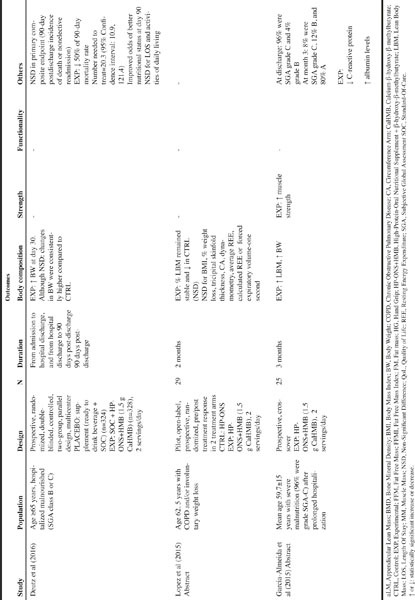
Summary of studies using HP-ONS+HMB in patients in peri-hospitalization setting

A multicenter randomized trial conducted by Malafarina et al. in 2017 (49) assessed whether HP-ONS+HMB improved muscle mass and nutritional markers in elderly patients with hip fracture. Ninety-two elderly patients (mean age 86 ±6 years) with hip fracture admitted to rehabilitation facilities received standard diet plus two servings per day of HP-ONS+HMB or standard diet only together with rehabilitation therapy (assisted mobility and physical exercise once a day for five days a week) for 30 days. Weight, muscle mass and appendicular lean mass (aLM) were stable in supplemented patients, whilst these parameters decreased in the control group, with a significant difference between groups (p<0.001 and p=0.020, respectively). Interestingly, fat mass did not change in both groups and more weight loss was observed in the control group (p<0.001). The predictive factors for Δ-aLM were ONS (p=0.006), Functional Ambulation Categories (FAC) score prior to fracture (p<0.001) and Barthel index (BI) prior to fracture (p=0.007). Supplemented patients showed higher intake of protein (p=0.007) and increased blood concentration of 25(OH)D (p<0.001) as compared to control group. In addition, greater percentage of supplemented patients showed functional recovery (68%) as compared to control group (59%), although the difference was not significant (p=0.265). This study concluded that supplementing the standard diet with a HP-ONS+HMB resulted in muscle mass preservation, and improved body composition in elderly patients with hip fracture admitted to rehabilitation unit.

Efficacy of HP-ONS+HMB combined with exercise has been assessed by Olveira et al (18) in non-malnourished non-cystic fibrosis bronchiectasis patients under pulmonary rehabilitation (n=30; aged 18–80 years). Here the effects of pulmonary rehabilitation with or without HP-ONS+HMB supplementation (1 serving/day) were compared over a 24 week period. Patients who received pulmonary rehabilitation plus HP-ONS+HMB showed improved FFM index at 12 weeks, and improved trunk FFM but not appendicular FFM at 12 and 24 weeks (p≤0.05). A significant increase from baseline was reported for bone mineral density (BMD), HGS (mean and maximal strength), mid-arm muscle circumference, and QoL (physical functioning scale) at both 12 and 24 weeks (p<0.01) in HP-ONS+HMB treated group. These results showed the benefits of the adjunctive use of a HP-ONS+HMB with an exercise rehabilitation program in improving strength and QoL in patients.

The effect of HP-ONS+HMB has been explored in other clinical outcomes together with muscle strength assessment. Conflicting results exist regarding the role of HP-ONS in hospital readmission [35] and mortality [34]. Few oral nutrition interventional studies exist in hospitalized populations with different pathologies aimed at evaluating impact on clinical outcomes such as LOS or mortality. While some systematic reviews have suggested that readmission and mortality can be significantly attenuated through using HP-ONS other systematic reviews and meta-analysis have not (33, 50). There are a number of reasons leading to inconsistencies in these studies namely low patients number, variability in the disease status of the patients studied or other important methodological limitations. The NOURISH (Nutrition effect On Unplanned Readmissions and Survival in Hospitalized patients) study represents one of the largest (n=622) RCT conducted so far in the nutrition field to evaluate readmission and survival. In this study Deutz et al. [19] explored the benefits of adding HP-ONS+HMB therapy to standard of care versus a placebo control added to standard of care alone on various outcomes such as hospital readmissions, nutritional indices, morbidity and mortality in malnourished, hospitalized older adults (≥ 65 years) with various comorbidities such as pneumonia, congestive heart failure, acute myocardial infarction, and COPD (4). Although no significant differences were detected between groups for the primary composite endpoint (90-day of first readmission and/or death and 90-day readmission rate), the 90-day mortality rate significantly decreased with HP-ONS+HMB when compared to the placebo group, 4.8% versus 9.7% (p=0.018), respectively, with a relative risk of 0.49 (95% confidence interval, 0.27 to 0.90). This lack of significant difference in readmission rate could be explained by the higher mortality rate in the placebo group, which could contribute to similar readmission rates observed in the 2 groups. In addition, while other efficacy variables such as mean total LOS and ADL were similar between treatments, the proportion of patients categorized as SGA-A (normally-nourished) increased over the study peaking at day 90 with 45.5% in the HP-ONS+HMB group compared with 30% in the placebo group.

An open-label prospective randomized study conducted by Lopez et al. (51) assessed the effect of a HP-ONS+HMB on muscle mass. HP-ONS+HMB was administrated to malnourished COPD patients (n=13; 62.5 years) and was compared to control patients who consumed a HP-ONS for 2 months. Results from this study indicated that the percentage change from baseline of LBM remained stable in the HP-ONS+HMB group and declined in the control group at the expense of fat mass, as measured by bioelectric impedance analysis. No statistical differences at month 1 and month 2 from baseline were detected for BMI, percentage of weight loss, tricipital skinfold thickness, circumference arm (CA), grip strength, average and calculated resting energy expenditure (REE) or forced expiratory volume-one second for both groups. Althought promising, the full publication of this study needs to be reported to critically assess the data.

A relationship between improved nutritional status and enhanced muscle function in malnourished hospitalized older patients has been also reported. In a recent published abstract, Garcia-Almeida et al. (49) conducted a prospective single arm, open label study in 50 adults (average age 59.7±15 years) with severe malnutrition (96% withgrade SGA-C), consuming 2 servings of HP-ONS+HMB for 3 months after prolonged hospitalization. This study showed improvements in their nutritional status such that the population on the whole moved from 96% SGA-C and 4% SGA-B upon hospitalization to 8% SGA-C, 12% SGA-B and 80% SGA-A upon discharge. A correlation between gains in lean mass and increased total energy intake diet was also observed, as well as increased serum albumin levels and improved muscle function at the end of study. Althought promising, the full publication of this study needs to be reported. Also, the lack of a placebo control and the open label design of this study warrents consideration when making conclusions on the benefits of the HP-ONS+HMB intervention in this study.

## Clinical relevance of HP-ONS+HMB

Although the number of publications are limited, there is an evolving literature pointing to the benefits of supplementing with HP-ONS+HMB to address muscle-related problems that develop with aging, chronic disease and/or immobilization, in addition to improving nutritional status especially in patients with or at risk of malnutrition. The combination of improving nutritional status along with muscle outcomes such as body composition, strength and mobility appear to translate to clinically relevant outcomes such as improved recovery time from hip surgery (21), improvements in the patients QoL (22) and even reduction in mortality (4). A couple of studies have combining exercise along with HP-ONS+HMB resulting in better clinical outcomes such as improved mobility (23), strength and QoL (22), than seen with just exercise alone, pointing to benefits of multimodal intervention whenever feasible. Importantly, HP-ONS+HMB formula also contains key ingredients that have been shown to significantly improve muscle-related outcomes such as calcium and vitamins D (52-55). The combination of all these micronutrients with HMB and other macronutrients such as high-quality protein levels seems to be a promising nutritional intervention to improve recovery of muscle mass and function in older adults.

There however remains a need for more well-controlled studies using HP-ONS+HMB in various clinical populations to identify the populations that will benefit the most from this specialized intervention.

## Concluding remarks

**Figure 2:**
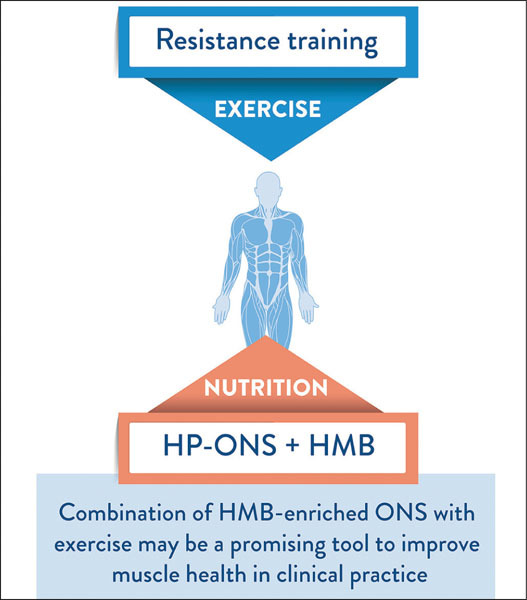
Lifestyle interventions to enhance muscle health

Overall, due to the scarce number of RCTs using ONS, limited evidence exists on the benefits of ONS in improving body composition and functionality in malnourished populations in different clinical settings. However, there is a cumulative body of evidence suggesting that a ‘therapeutic’ ONS enriched with specific nutrients could have a positive impact on adults under catabolic conditions, especially among hospitalized patients. These specific nutrients such as HMB have shown promising effects on maintenance of muscle mass and strength in various clinical settings. It has been suggested that HP-ONS+HMB may be a promising nutritional approach to maintain or improve muscle mass and function in adults under different health and disease conditions. Furthermore, in hospitalized patients, supplementation with a HP-ONS+HMB formula was linked to significant improvement in patient-related clinical outcomes. Therefore, combination of HP-ONS+HMB with standard of care programs may be a promising tool to improve muscle health in clinical practice, especially during patient rehabilitation and recovery (Fig. 2). Nevertheless, there is still a need for more RCTs using HP-ONS+HMB administration in various clinical settings to fully understand the scope of the benefits that supplementation could provide under various catabolic conditions.

*Author Contributions:* A.S.P. formulated the review topic. M.C.R performed the literature review and A.S.P, M.C.R, S.L.P and A.J.C.J. wrote the manuscript. J.M.L.P., R.R.C., M.D.B.P. and J.M.G.A. revised the manuscript and M.C.R. edited drafts of this review. All authors read and approved the final manuscript.

*Conflicts of Interests:* M.C.R., J.M.L.P., S.L.P. and R.R. are employed by Abbott Nutrition. A.J.C.J. has received speaker and research funds from Abbott Nutrition.

*Ethical standard:* I declare that the review comply with the current laws of the country in which it was performed”

## References

[1] Shiozu H, Higashijima M, Koga T (2015). Association of sarcopenia with swallowing problems, related to nutrition and activities of daily living of elderly individuals. J Phys Ther Sci..

[2] Argiles JM, Campos N, Lopez-Pedrosa JM, Rueda R, Rodriguez-Manas L (2016). Skeletal Muscle Regulates Metabolism via Interorgan Crosstalk: Roles in Health and Disease. J Am Med Dir Assoc..

[3] de Souza Vasconcelos KS, Domingues Dias JM, de Carvalho Bastone A, Alvarenga Vieira R, de Souza Andrade AC, Rodrigues Perracini M (2016). Handgrip Strength Cutoff Points to Identify Mobility Limitation in Community-dwelling Older People and Associated Factors. J Nutr Health Aging..

[4] Deutz NE, Matheson EM, Matarese LE, Luo M, Baggs GE, Nelson JL (2016). Readmission and mortality in malnourished, older, hospitalized adults treated with a specialized oral nutritional supplement: A randomized clinical trial. Clin Nutr..

[5] Cruz-Jentoft AJ, Baeyens JP, Bauer JM, Boirie Y, Cederholm T, Landi F (2010). Sarcopenia: European consensus on definition and diagnosis: Report of the European Working Group on Sarcopenia in Older People. Age Ageing..

[6] Kamel HK (2003). Sarcopenia and aging. Nutr Rev..

[7] Hirani V, Blyth F, Naganathan V, Le Couteur DG, Seibel MJ, Waite LM (2015). Sarcopenia Is Associated With Incident Disability, Institutionalization, and Mortality in Community-Dwelling Older Men: The Concord Health and Ageing in Men Project. J Am Med Dir Assoc..

[8] Elia M, RJ S (2009). Calculating the cost of disease-related malnutrition in the UK in 2007 (public expenditure only) in: Combating Malnutrition: Recommendations for Action. Report from the advisory group on malnutrition.

[9] Scott D, Park MS, Kim TN, Ryu JY, Hong HC, Yoo HJ (2016). Associations of low muscle mass and the metabolic syndrome in Caucasian and Asian middle-aged and older adults. J Nutr Health Aging..

[10] Greysen SR, Stijacic Cenzer I, Auerbach AD, Covinsky KE (2015). Functional impairment and hospital readmission in Medicare seniors. JAMA Intern Med..

[11] Guest J P, Baeyens JP, de Man F, Ljungqvist O, Pichard C, Wait S, Wilson L (2011). Health economic impact of managing patients following a community-based diagnosis of malnutrition in the UK. Clin Nutr..

[12] Kaiser MJ, Bauer JM, Rämsch C, Uter W, Guigoz Y, Cederholm T (2010). Frequency of malnutrition in older adults: a multinational perspective using the mini nutritional assessment. J Am Geriatr Soc..

[13] Cruz-Jentoft AJ, Landi F, Schneider SM, Zuniga C, Arai H, Boirie Y (2014). Prevalence of and interventions for sarcopenia in ageing adults: a systematic review. Report of the International Sarcopenia Initiative (EWGSOP and IWGS). Age Ageing..

[14] Wandrag L, Brett SJ, Frost G, Hickson M (2015). Impact of supplementation with amino acids or their metabolites on muscle wasting in patients with critical illness or other muscle wasting illness: a systematic review. J Hum Nutr Diet..

[15] Witard OC, Wardle SL, Macnaughton LS, Hodgson AB, Tipton KD (2016). Protein Considerations for Optimising Skeletal Muscle Mass in Healthy Young and Older Adults. Nutrients.

[16] Landi F, Calvani R, Tosato M, Martone AM, Ortolani E, Savera G (2016). Protein Intake and Muscle Health in Old Age: From Biological Plausibility to Clinical Evidence. Nutrients.

[17] Molfino A, Gioia G, Rossi Fanelli F, Muscaritoli M (2013). Beta-hydroxy-beta-methylbutyrate supplementation in health and disease: a systematic review of randomized trials. Amino Acids..

[18] Standley RA, Distefano G, Pereira SL, Tian M, Kelly OJ, Coen PM (1985). Effects of beta-hydroxy-beta-methylbutyrate (HMB) on skeletal muscle mitochondrial content and dynamics, and lipids after 10 days of bed rest in older adults. J Appl Physiol.

[19] Fitschen PJ, Wilson GJ, Wilson JM, Wilund KR (2013). Efficacy of beta-hydroxy-betamethylbutyrate supplementation in elderly and clinical populations. Nutrition..

[20] Cruz-Jentoft AJ (2017). Beta-hydroxy-beta-methyl butyrate (HMB): From experimental data to clinical evidence in sarcopenia. Curr Protein Pept Sci..

[21] Ekinci O, Yanik S, Terzioglu Bebitoglu B, Yilmaz Akyuz E, Dokuyucu A, Erdem S (2016). Effect of Calcium beta-Hydroxy-beta-Methylbutyrate (CaHMB), Vitamin D, and Protein Supplementation on Postoperative Immobilization in Malnourished Older Adult Patients With Hip Fracture: A Randomized Controlled Study. Nutr Clin Pract..

[22] Olveira G, Olveira C, Dona E, Palenque FJ, Porras N, Dorado A (2016). Oral supplement enriched in HMB combined with pulmonary rehabilitation improves body composition and health related quality of life in patients with bronchiectasis (Prospective, Randomised Study). Clin Nutr..

[23] Berton L, Bano G, Carraro S, Veronese N, Pizzato S, Bolzetta F (2015). Effect of Oral Beta-Hydroxy-Beta-Methylbutyrate (HMB) Supplementation on Physical Performance in Healthy Old Women Over 65 Years: An Open Label Randomized Controlled Trial. PLoS One..

[24] Philipson TJ, Snider JT, Lakdawalla DN, Stryckman B, Goldman DP (2013). Impact of oral nutritional supplementation on hospital outcomes. Am J Manag Care..

[25] Collins J, Porter J (2015). The effect of interventions to prevent and treat malnutrition in patients admitted for rehabilitation: a systematic review with meta-analysis. J Hum Nutr Diet..

[26] Graham J, Fan L, Meadows ES, Hang L, Partridge J, Goates S (2017). Addressing malnutrition across the continuum of care: Which Patients Are Likely to Receive Oral Nutritional Supplements. Journal of ageing research and healthcare.

[27] Sauer AC, Alish CJ, Strausbaugh K, West K B Q (2016). Nurses needed: Identifying malnutrition in hospitalized older adults. NursingPlus Open..

[28] Schuetz P (2017). Food for thought: why does the medical community struggle with research about nutritional therapy in the acute care setting. BMC Med..

[29] Baldwin C, Weekes CE (2012). Dietary counselling with or without oral nutritional supplements in the management of malnourished patients: a systematic review and meta-analysis of randomised controlled trials. J Hum Nutr Diet..

[30] Bally MR, Blaser Yildirim PZ, Bounoure L, Gloy VL, Mueller B, Briel M (2016). Nutritional Support and Outcomes in Malnourished Medical Inpatients: A Systematic Review and Meta-analysis. JAMA Intern Med..

[31] Elia M, Normand C, Laviano A, Norman K (2016). A systematic review of the cost and cost effectiveness of using standard oral nutritional supplements in community and care home settings. Clin Nutr..

[32] Elia M, Normand C, Norman K, Laviano A (2016). A systematic review of the cost and cost effectiveness of using standard oral nutritional supplements in the hospital setting. Clin Nutr..

[33] Stratton RJ, Hebuterne X, Elia M (2013). A systematic review and meta-analysis of the impact of oral nutritional supplements on hospital readmissions. Ageing Res Rev..

[34] Milne AC, Potter J, Vivanti A, Avenell A (2009). Protein and energy supplementation in elderly people at risk from malnutrition. Cochrane Database Syst Rev..

[35] Cawood AL, Elia M, Stratton RJ (2012). Systematic review and meta-analysis of the effects of high protein oral nutritional supplements. Ageing Res Rev..

[36] Wall BT, van Loon LJ (2013). Nutritional strategies to attenuate muscle disuse atrophy. Nutr Rev..

[37] Katsanos CS, Kobayashi H, Sheffield-Moore M, Aarsland A, Wolfe RR (2005). Aging is associated with diminished accretion of muscle proteins after the ingestion of a small bolus of essential amino acids. Am J Clin Nutr..

[38] Deutz NE, Bauer JM, Barazzoni R, Biolo G, Boirie Y, Bosy-Westphal A (2014). Protein intake and exercise for optimal muscle function with aging: recommendations from the ESPEN Expert Group. Clin Nutr..

[39] Koretz RL, Avenell A, Lipman TO, Braunschweig CL, Milne AC (2007). Does enteral nutrition affect clinical outcome? A systematic review of the randomized trials. Am J Gastroenterol..

[40] Wu H, Xia Y, Jiang J, Du H, Guo X, Liu X (2015). Effect of beta-hydroxy-betamethylbutyrate supplementation on muscle loss in older adults: a systematic review and meta-analysis. Arch Gerontol Geriatr..

[41] Flakoll P, Sharp R, Baier S, Levenhagen D, Carr C, Nissen S (2004). Effect of beta-hydroxybeta-methylbutyrate, arginine, and lysine supplementation on strength, functionality, body composition, and protein metabolism in elderly women. Nutrition..

[42] Stout JR, Smith-Ryan AE, Fukuda DH, Kendall KL, Moon JR, Hoffman JR (2013). Effect of calcium beta-hydroxy-beta-methylbutyrate (CaHMB) with and without resistance training in men and women 65+yrs: a randomized, double-blind pilot trial. Exp Gerontol..

[43] Nishizaki K, Ikegami H, Tanaka Y, Imai R, Matsumura H (2015). Effects of supplementation with a combination of beta-hydroxy-beta-methyl butyrate, L-arginine, and L-glutamine on postoperative recovery of quadriceps muscle strength after total knee arthroplasty. Asia Pac J Clin Nutr..

[44] Kortebein P, Symons TB, Ferrando A, Paddon-Jones D, Ronsen O, Protas E (2008). Functional impact of 10 days of bed rest in healthy older adults. J Gerontol A Biol Sci Med Sci..

[45] Deutz NE, Pereira SL, Hays NP, Oliver JS, Edens NK, Evans CM (2013). Effect of betahydroxy- beta-methylbutyrate (HMB) on lean body mass during 10 days of bed rest in older adults. Clin Nutr..

[46] De Luis DA, Izaola O, Bachiller P, Perez Castrillon J (2015). Effect on Quality of Life and Handgrip Strength by Dynamometry of an Enteral Specific Suplements with Beta-Hydroxy-Beta-Methylbutyrate and Vitamin D in Elderly Patients. Nutr Hosp..

[47] Cramer JT, Cruz-Jentoft AJ, Landi F, Hickson M, Zamboni M, Pereira SL (2016). Impacts of High-Protein Oral Nutritional Supplements Among Malnourished Men and Women with Sarcopenia: A Multicenter, Randomized, Double-Blinded, Controlled Trial. J Am Med Dir Assoc..

[48] Kuhls DA, Rathmacher JA, Musngi MD, Frisch DA, Nielson J, Barber A (2007). Beta-hydroxy-beta-methylbutyrate supplementation in critically ill trauma patients. J Trauma..

[49] Malafarina V, Uriz-Otano F, Malafarina C, Martinez JA, Zulet MA (2017). Effectiveness of nutritional supplementation on sarcopenia and recovery in hip fracture patients. A multi-centre randomized trial. Maturitas..

[50] Beck AM, Holst M, Rasmussen HH (2013). Oral nutritional support of older (65 years+) medical and surgical patients after discharge from hospital: systematic review and metaanalysis of randomized controlled trials. Clin Rehabil..

[51] Lopez JA, Segurola H, Cardenas G, Giribes M, Ferrer JJ, Rodriguez E (2015). Effect on muscle mass of nutritional intervention with a hypercaloric and hyperproteic diet with HMB in malnourished patients with COPD. Nutr Hosp..

[52] Tu MK, Levin JB, Hamilton AM, Borodinsky LN (2016). Calcium signaling in skeletal muscle development, maintenance and regeneration. Cell Calcium..

[53] Halfon M, Phan O, Teta D (2015). Vitamin D: a review on its effects on muscle strength, the risk of fall, and frailty. Biomed Res Int..

[54] Bischoff-Ferrari HA, Dawson-Hughes B, Staehelin HB, Orav JE, Stuck AE, Theiler R (2009). Fall prevention with supplemental and active forms of vitamin D: a meta-analysis of randomised controlled trials. BMJ.

[55] Bischoff-Ferrari HA, Giovannucci E, Willett WC, Dietrich T, Dawson-Hughes B (2006). Estimation of optimal serum concentrations of 25-hydroxyvitamin D for multiple health outcomes. Am J Clin Nutr..

